# Unfolding the science behind policy initiatives targeting plastic pollution

**DOI:** 10.1186/s43591-022-00046-y

**Published:** 2023-02-02

**Authors:** Maria Bille Nielsen, Lauge Peter Westergaard Clausen, Richard Cronin, Steffen Foss Hansen, Nikoline Garner Oturai, Kristian Syberg

**Affiliations:** 1 Department of Environmental and Resource Engineering, Technical University of Denmark, Kongens Lyngby, Denmark; 2 Marine Environment Section, Department of Housing, Local Government and Heritage, Wexford, Ireland; 3 Department of Science and Environment, Roskilde University, Roskilde, Denmark

**Keywords:** Plastics pollution, Policy initiatives, Better regulation, Risks, Scientific evidence, Uncertainty

## Abstract

The intensive global plastic production, use and associated plastic pollution have caused concern for the potential risks to human health and the environment. This has led to the adoption of numerous regulatory initiatives aiming to combat plastic pollution. Despite the considerable regulatory activity in the field of plastic, it appears that there is still debate about the actual risks of plastic to humans and the environment. This raises the question of to what extent the current plastic regulation is evidence-based, a declared ambition in the European Union. Therefore, the aim of this study was to investigate to what extent key policy initiatives targeting plastic pollution are based on scientific evidence. Selection of initiatives was based on expert elicitation accounting for the opinions of persons involved in the development of the policy initiatives, and a thorough assessment of the historical development of plastic pollution regulation, with focus on their importance both with respect to regulation of plastics as well as their historical importance as drivers for societal actions on plastic pollution. We find that scientific evidence appears to be generally present in the scientific foundation for the policy initiatives analysed in this study. All the initiatives are supported by scientific articles and reports about among others plastic sources, ecological impacts of plastic production and consumption patterns. Marine litter monitoring data was found to contribute to the evidence base for 4 out of the 6 policy initiatives and thereby appears to be one of the central scientific drivers behind the societal actions on plastic pollution. Other scientific tools applied when shaping the policy initiatives include risk assessment, impact assessment and life cycle assessment. Despite the prevalent consideration and application of scientific evidence, there seems to be a broad recognition in the preparatory work of the initiatives that there is still a lot of uncertainty related to determining the harm of plastic pollution. In these cases, taking precautionary actions seems however to be justified, recalling not least the precautionary principle. As the issue of plastic pollution is complex and still subject to uncertainty, it seems important both that policy initiatives allow for flexibility and continuing adjustment to the on-going knowledge generation and that the scientific community provides the needed research to continue the science-informed policy development.

## Introduction

The widespread use of plastics globally and the associated plastic pollution have caused concern for potential risks to human health and the environment, and received increasing attention from citizens, media, policy makers and the scientific community [1]. This has led to the adoption of numerous regulatory initiatives aiming to combat plastic pollution both at the national and international level. Some of the first initiatives to target plastic pollution were taken by United National Environmental Assembly (UNEA) through the adoption of the resolution on 'Marine plastic debris and microplastics' in 2014 as well as the OSPAR and HELCOM regional action plans targeting marine litter, adopted in 2014 and 2015, respectively [2–4], with discussions going back approximately 10 years before this. Already in 2008, the so-called “ghost net paper” was published by Bord Iascaigh Mhara (BIM) and Norwegian experts, which provided some of the first evidence that significant amounts of lost fishing gear were present in the coastal areas of Rockall and Porcupine Bank [5]. In 2013, the European Commission’s (EC) news alert service Science for Environment Policy stated that plastics debris is both a physical and chemical pollutant of “serious environmental concern” [6]. The EC’s statement was based on a recent study by Rochman et al. [7] that found sorption of contaminants (polychlorinated biphenyls and polycyclic aromatic hydrocarbons) to plastics. The OSPAR and HELCOM action plans are closely linked to the adoption of descriptor 10 on prevention of marine litter in the European Marine Strategy Framework Directive (MSFD) [3, 8] and constitute an essential historical driver for regulations targeting plastic pollution. Descriptor 10 is one out of 11 qualitative descriptors that form the basis for the determination of good environmental status and it states “Properties and quantities of marine litter do not cause harm to the coastal and marine environment” [8]. Several important policy initiatives followed in the years after, including the European Union’s (EU’s) Directive amendment regarding lightweight plastics carrier bags [9], EU’s Strategy for Plastics in a Circular Economy [10], the amendment of the Basel Convention regarding plastics waste [11], EU Single Use Plastics (SUP) Directive [12] and EC’s proposal on restriction of intentionally added microplastics [13].

Despite the considerable regulatory activity in the field of plastics, it appears that there is still debate about the actual risks of plastics to humans and the environment [14, 15]. Numerous studies have demonstrated impacts by plastics on both humans and wildlife (e.g., [16–18]) as well as on ecosystem services [19]. Still, as an example, Burton [20] argued that focus on microbeads risk by the scientific community was harmful, since the risk was superficial as a consequence of the low microplastic exposure concentrations. This initiated a debate concerning the necessity to document risk as the best scientific foundation for societal actions (e.g., [21, 22]). A recent report from Science Advice for Policy by European Academies (SAPEA) reviewed the current evidence on health, environmental and societal impacts of nano- and microplastics pollution and also here it was concluded that little is still known about the risks and that “what is known is surrounded by considerable uncertainty” [14].

The Covid-19 pandemic has revitalized the use of single-use plastic products [23]. Still some stakeholders have argued that restricting the use of such plastic products would not be the best solution from an environmental perspective when taking compensatory behaviour into account [24]. The plastics industry association in New Zealand, Plastics NZ, has also expressed their concern about targeting only plastic packaging alone, while not focusing on packaging materials in general [25]. Finally, it has been argued that the intensive focus on plastic pollution is drawing attention away from more important problems such as climate change and the impact on biodiversity from overfishing [26]. This raises the question of to what extent the current plastic regulation is evidence-based, a declared ambition in the EU since the beginning of the 2000’s [27, 28].

Recently, in April 2021, the EC adopted their “Communication on Better Regulation*”* which sets out a list of improvement proposals to EU law-making [29, 30]. According to the communication, one of the key principles for ‘Better regulation’ is the use of scientific evidence to describe the problem, understand causality and intervention logic and lastly to assess impact. To do this, the research community must be mobilized and engaged early in the regulatory process [29]. According to the EC’s ‘Better Regulation’ toolbox scientific evidence is defined as data, information, and knowledge from multiple sources, including statistics, measurements, stakeholder input and scientific and expert advice. Importantly, in order for evidence to be considered reliable it must be based on “the appropriate method to collect, interpret, process and transform data and information” [30].

As much is still unknown about the environmental impacts of plastic pollution, it may be challenging to provide fully comprehensive evidence-based policies. While uncertainty should not hinder political action, recalling that the precautionary principle of the UN Rio Declaration from 1992 explicitly states that uncertainty should not be used as an excuse for not implementing regulatory action [31], exploring the quality and nature of the scientific evidence behind the political initiatives may uncover important aspects of the role science plays and has played in guiding policy actions targeting plastic pollution. Here, we therefore address the research question “what is the scientific underpinning of plastic pollution policies in a historical context”. To explore this the aim of this study is to investigate to what extent key policy initiatives targeting plastic pollution are based on scientific evidence and what types of evidence have been used. The focus is on policy initiatives aiming to reduce the environmental pollution of plastics originating from the production, use and end-of-life handling of plastics. Furthermore, we examine whether the knowledge base which regulations rely on consider “state of the art” and discuss the different types of scientific evidence and tools used to guide policy actions targeting environmental plastic pollution.

## Methods

In order to investigate the use of scientific evidence in shaping key policy initiatives targeting plastic pollution, a list of key initiatives was identified and selected. Apart from experts in the author team, a dialogue with external experts were used to obtain the needed understanding of the policy processes. Selection of initiatives were thus based on expert elicitation accounting for the opinions of persons involved in the development of the policy initiatives, and a thorough assessment of the historical development of plastic pollution regulation, with focus on their importance both with respect to regulation of plastics as well as their historical importance as drivers for societal actions on plastic pollution. As mentioned in the introduction, the OSPAR and HELCOM action plans on marine litter constitute an important historical driver for policy initiatives aiming to combat plastic pollution, as some of the first initiatives putting focus on marine litter, including plastics litter. The OSPAR and HELCOM action plans were therefore selected to be part of the analysis. Due to the parallels and similarities between the OSPAR and HELCOM Action Plans (and their associated sister conventions), the OSPAR Action Plan will be representative for both. Another policy initiative included in the analysis is the amendments of the Basel Convention regarding plastic waste [11]. With the amendments, adopted in 2019, the Basel Convention became the first initiative to specifically address plastic waste [32]. Raubenheimer & McIlgorm [33] pointed to a list of limitations of this treaty’s amendments, however, still argued that the Basel Convention has the potential to reduce the impacts of plastics globally. Four EU policy initiatives were selected to be part of the analysis, which all play an important role in targeting plastic pollution. The focus on EU in this context stems from the fact that EU aims at being a driver for transition to circular economy, and have a suit of regulations aiming to facilitate this. Analyzing these in a common context thus provide a more rigor foundation for understanding the science behind. The first is the EU’s Directive amendment regarding lightweight plastics carrier bags [9]. The Directive amendment, adopted in 2015, was the first initiative to target plastic bags at European level. The second EU initiative included in this study is the EU’s Strategy for Plastics in a Circular Economy [10]. According to Palm et al. [34], this strategy forms the basis of the EU’s approach to plastics governance for the next decade and constitutes a significant milestone in the global debate about how to solve the plastics issue. Another important EU initiative selected for this analysis, is the EU Single Use Plastics Directive [12]. This Directive is considered the most comprehensive regulation combatting plastic pollution worldwide [35]. It introduces, among others, bans on certain single-use plastics products, Extended Producer Responsibility schemes and awareness-raising measures to reduce consumption, all of which have been highlighted as important elements for efficient plastics management and governance [36]. Finally, the EC’s proposal on restriction of intentionally added microplastics [13] – the most comprehensive restriction action in the world for reducing intentional microplastic use emissions [37]. 

The scientific foundation for each of the selected initiatives were assessed by first of all identifying the preparatory work to each of the initiatives. The preparatory work was identified by analyzing the main documents presenting the initiatives (legal documents, strategy descriptions, action plan descriptions etc.) and via semi-structured searches of webpages and databases of the organisations/institutions/commissions behind the initiatives. When the preparatory work was identified, it was analysed for its inclusion of and reference to scientific evidence. In this study, scientific evidence is defined according to the EC’s ‘Better Regulation’ toolbox [38]. The ‘Better Regulation’ toolbox sets out principles to be used when preparing new policy initiatives and includes principles for evidence-informed policymaking. In the principles for evidence-informed policymaking it is described that the term ‘evidence’ refers to “data, information, and knowledge from multiple sources, including quantitative data such as statistics and measurements, qualitative data such as opinions, stakeholder input, conclusions of evaluations, as well as scientific and expert advice.” It is further noted, that for evidence to be reliable it must be based on “the appropriate method to collect, interpret, process and transform data and information” [38]. In the following, the scientific evidence for the six selected policy initiatives targeting plastic pollution is presented. The findings are presented chronologically.

## Results

### Scientific foundation for the OSPAR Action Plan on Marine Litter

In March 1998, the Convention for the Protection of the Marine Environment of the North-East Atlantic (OSPAR Convention) [39]—the legal instrument managing the international collaboration when it comes to the protecting the marine environment in the area—entered into force. It aims to protect the parts of the Atlantic and Arctic Oceans lying north of 36° north latitude and between 42 west longitude and 51° east longitude and the part of the Atlantic Ocean north of 59° north latitude and between 44° west longitude and 42° west longitude [40]. In relation to marine litter, OSPAR set out an objective to “substantially reduce marine litter in the OSPAR Maritime Area to levels where properties and quantities do not cause harm to the marine environment” [41]. In 2010 the OSPAR Contracting Parties committed themselves to the North-East Atlantic Environment Strategy (NEAES) 2010–2020 with their Bergen Statement [42] which obligates to “develop appropriate programmes and measures to reduce amounts of litter in the marine environment and to stop litter entering the marine environment, both from sea-based and land-based sources” [41]. To accomplish the aim of the NEAES, the Regional Action Plan for Marine Litter (2014–2021) (hereafter OSPAR Action Plan) was adopted in 2014. The OSPAR Action Plan includes a list of national and collective actions which fall into the following four themes: actions to combat sea-based sources of marine litter, actions to combat land-based sources of marine litter; marine litter removal actions, and actions for marine litter education and outreach. The predominant part of the actions has a large-scale and transboundary character and thereby applies at a regional level. The OSPAR Action Plan is supportive of Descriptor 10 in the MSFD [3]. Already by 2009 OSPAR recognised that its work on marine litter had synergies with the EU MSFD Task Group 10 for the Litter Descriptor [43] and also the EC noted that implementation of the MSFD is closely linked to the OSPAR Convention and the other regional sea conventions [44]. In the following, the scientific foundation for the OSPAR Action Plan will be described.

As part of the development of the OSPAR Action Plan, a series of workshops were held. According to the terms of reference on a workshop on the development of an OSPAR Regional Action Plan on Marine Litter from 2013 [45], the workshop took basis on the following reports: the Checklist developed by the OSPAR Coordination Group (CoG) in 2012 [46], the United Nation Environmental Programme (UNEP) Issue Paper from the International Conference on Prevention and Management of Marine Litter in European Seas [47], the Quality Status Report 2010 [48], and its underlying assessments, the results for the OSPAR Beach Litter Monitoring [49, 50] and the Fulmar Ecological Quality Objectives (EcoQO) [48, 51]. In addition to these reports, the workshop further considered “current scientific knowledge and monitoring results.” A questionnaire was also distributed to the Contracting Parties with the purpose to collect supplementary information on monitoring, marine litter sources and opinions regarding choice of measures and targets [3].

The aim of the UNEP Issue Paper [47] was to provide a common starting point for the participants at the International Conference on Prevention and Management of Marine Litter in European Seas held in Berlin April 2013. The conference’s goal was to further develop the regional action plans on marine litter and regional sea conventions in European waters. The document contains among others “up-to-date knowledge” (at that time) on marine litter impacts, presence and sources and overall principles of combating marine litter. When it comes to impacts of plastic debris, this knowledge is among others based on reports authored by MSFD Good Environmental Status Technical Group on Marine Litter (TG-ML) from 2011, published by the Joint Research Centre (JRC) [52], and the UNEP, Secretariat of the Convention on Biological Diversity [53]. The reports describe that it seems inevitable that entanglement and ingestion of plastic debris will alter biological and ecological performance of individuals. Negative effects include food digestion inabilities, decrease in reproduction and body condition and facilitation of transport of harmful chemicals. A lot of scientific literature is also cited, documenting *inter alia* observations of plastics entanglement by species such as sharks and seals, plastics ingestion by whales and degradation of coral reefs by fishing gear. When it comes to the sources and amounts of plastic litter, the information includes data from the UNEP [54] and the consultancy organization ARCADIS [55], as well as different scientific papers published in the period from 1997 to 2013. It also includes data from reports from the OSPAR Commission, including their report on monitoring of marine litter in the OSPAR region from 2007, and according to the report it is “based on present knowledge base.” A list of guiding principles that should provide a framework for action on marine litter is highlighted in the report. The principle of prevention at source, the precautionary principle and principle of polluter-pays are listed with reference to their enshrinement in Article 191(2) in the Treaty on the Functioning of the European Union (TFEU) and application in e.g., Article 2.2 of the OSPAR and Helsinki Conventions. The ecosystem-based approach is also listed with reference to its confirmation in the Conference to the Parties of the Convention of Biological Diversity, Johannesburg Plan of Implementation and the Rio + 20 Conference.

The Issue Paper puts forward guidance on how to set environmental targets for the different components of the marine water [47]. The paper further describes the framework for actions to combat plastic litter in the European marine waters and applies in that connection primarily the book Tackling Marine Debris in the 21^st^ Century by the National Research Council (NRC), Committee on the Effectiveness of International and National Measures to Prevent and Reduce Marine Debris and its Impact [56] and report from UNEP of 2009 called Marine Litter: A Global Challenge.

The Quality Status Report 2010 [48] published by the OSPAR Commission presents the status of the North-East Atlantic in 2010 and how it has changed since 2000. The report was drafted by scientists and experts from the OSPAR Contracting Parties and observer organisations and was peer-reviewed by a group of international scientists. The report stresses concern about the presence of marine litter, especially plastic, and builds on two assessments; one on an OSPAR pilot project on marine beach litter monitoring [57] and one on the assessment of marine litter in the North-East Atlantic Region [58].

The OSPAR Checklist on Marine Litter (Annex III, [47]) consist of possible strategic directions to be considered to prevent litter from causing harm in the North-East Atlantic. It was drafted by the Inter-sessional Correspondence Group on Marine Litter (ICG-ML), originally made with the intention to construct the OSPAR Action Plan. The Checklist does not point to specific scientific knowledge in itself, but describes that the proposed strategies should be considered and evaluated in light of current knowledge regarding predominant sources, composition and amounts of marine litter, cost effectiveness, effects on the internal market, legal feasibility, existing policies and legislation, as well as knowledge regarding the impacts of marine litter within the regional and sub-regional ecosystems.

Marine litter monitoring is an important part of the work done by OSPAR [49] and has taken place since 2001 [59]. In 2010 the OSPAR Commission published their report on guidance for monitoring of marine beach litter in the OSPAR region [50]. It uses an approach developed in an OSPAR pilot project with supplements from UNEPs “realization of a worldwide guideline.” The guidelines are constructed so all the OSPAR Contracting Parties can take part in the monitoring, while ensuring sufficient data quality [50].

The EcoQOs system for the North Sea defines “the desired qualities of a component of the ecosystem” and has been developed since 1992 by the OSPAR Commission and the International Council for the Exploration of the Sea (ICES) [48]. In 2002, the OSPAR Commission included marine plastic litter in the EcoQOs system. At that time, the Netherlands was monitoring marine litter by use of the Northern Fulmar and the abundance of plastics in its stomach, as indicator. This monitoring approach was adopted by OSPAR as one of its EcoQOs and it was set as a long-term goal that “There should be less than 10% of Northern fulmars (*Fulmarus glacialis*) having 0.1 g or more plastics in the stomach in samples of 50–100 beached fulmars from each of 5 different areas of the North Sea over a period of at least 5 years.” The monitoring approach looks at fulmars that are found dead or accidentally killed [51, 60].

In OSPAR’s Quality Status Report from 2010 [50], which is part of the science underpinning the OSPAR Action plan, it is stated that the ecological effects of marine litter are still not completely known. Also, the UNEP Issue Paper [47] points to some uncertainties, e.g., including differences in reporting frequencies when it comes to plastics ingestion and entanglement data, potentially leading to interpretation biases. Additionally, UNEP [47] identifies a list of knowledge gaps when it comes to description of marine litter sources, which cover amounts, composition, transport, origin and impacts of marine litter in the water column, amounts, sources and impacts of microplastics, and contribution of marine litter from rivers. It is further noted that when considering “number of [plastic] items”, the importance of the items’ impact is not automatically represented. As previously described, UNEP [47] explicitly refers to the consideration of the precautionary principle, thereby justifying precautionary actions despite of uncertainty.

### Scientific foundation for the European Directive amendment on lightweight plastic carrier bags

In 2015, the European Parliament and the Council adopted Directive 2015/720/EU amending Directive 94/62/EC on packaging and packaging waste as regards the consumption of lightweight plastic carrier bags [9]. The Directive was further amended in 2018 to account for the green transition to a more circular economy [61]. The Directive requires Member States to take measures to achieve a sustained reduction in the consumption of lightweight plastic carrier bags. “Lightweight plastic carrier bags” are defined as plastic carrier bags with a wall thickness below 50 microns. The measures to be used to achieve this objective are up to the individual Member States and may include the use of national reduction targets, maintaining or introducing economic instruments as well as marketing restrictions. They shall, however, include either or both 1) the adoption of measures to ensure that the annual consumption level does not exceed 90 and 40 lightweight plastic carrier bags per capita by 2020 and 2026 respectively; and 2) the adoption of instruments ensuring that lightweight plastic carrier bags are not provided free of charge at the point of sale before 2019, unless equally effective instruments are implemented. The Directive requires the Commission to lay down the methodology for the calculation of the annual consumption of lightweight plastic carrier bags per capita and that Member States report this consumption annually. The Commission and Member States must also actively encourage public information and awareness campaigns about the adverse environmental impact of the excessive consumption of lightweight plastic carrier bags. Finally, the Commission will lay down specific measures on labelling of biodegradable and compostable plastic carrier bags to ensure Union-wide recognition and to provide consumers with the correct information about the composting properties of such bags.

The proposal for amending Directive 94/62/EC was initiated by the EC in 2013 [6]. As the basis of the proposed amendments and under the heading “Consultation and expertise,” the Commission refers to studies from 2011 and 2012 on the production and consumption patterns of plastic carrier bags, their impacts and the impacts of different policy options to reduce their use was performed and a study to assess the socio-economic impacts of different policy options.

The Commission’s proposal was subject to an impact assessment (IA) and socio-economic analysis. These were prepared by BioIntelService [62] and Eunomia [63], respectively. With regard to the environmental aspects of plastic carrier bags, a life-cycle assessment (LCA) prepared by the UK Environment Agency [64] is used by BioIntelService [62] to conclude that the “environmental impacts of plastic carrier bags over their life cycle depend on their thickness, whether and how often they are reused and what happens to them at end of life” [62]. The LCA by the UK Environment Agency considered the impacts from the number of bags required to carrying one month’s shopping in 2006/07. It was calculated how many times each different type of carrier would have to be used to reduce its global warming potential to below that for conventional High Density Poly Ethylene (HDPE) carrier bags where some 40% were reused as bin liners. The carriers were furthermore compared to other impacts: Resource depletion, acidification, eutrophication, human toxicity, fresh water aquatic ecotoxicity, marine aquatic ecotoxicity, terrestrial ecotoxicity and photochemical oxidation (smog formation). It was found that the environmental impact of all types of carrier bags is dominated by resource use in the production stages whereas transport, secondary packaging and end-of-life management was found to have minimal influence on their performance. Inventory data used to complete the LCA was primarily taken from the ecoinventTM database version 2. In the case of HDPE, low density polyethylene (LDPE), linear low density polyethylene (LLDPE) and polypropylene, PlasticsEurope datasets cover the production from cradle to the polymer factory gate [64]. In the IA by BioIntelService [62], the LCA is supplemented by references to one article published in Marine Pollution Bulletin in 2002 by Derraik [65] as well as several articles published in 2009 and 2010 in Philosophical Transactions of the Royal Society B: Biological Sciences on Plastics by e.g. Thomson et al. [66] and Barnes et al. [67].

Scientific uncertainties in relation to the LCA within BioIntelService’s IA for EC are addressed as dominated by factors that were “downstream of the shopping bag manufacturing (consumer behavior, landfill conditions, method of waste combustion, etc.)” [62, 64]. Furthermore, the incompleteness of available data along with the complexity of the issues for creating the baseline scenario made for further uncertainties [62]. Eunomia [63] accounts for uncertainties in relation to the challenge of estimating counts and rates of plastic bags already in the environment (referred to as “stocks”); “Therefore, while estimates of the rate of littering of plastic bags by Member State are highly uncertain, there is even greater uncertainty in the impact that this may have on the total costs of cleaning up litter (due to the existence of stocks [..])” [63].

### Scientific foundation for the European Strategy for Plastics in a Circular Economy

In 2018, the EC presented their European Strategy for Plastics in a Circular Economy [10], which aimed at laying the foundations to a new plastics economy, where the design and production of plastics and plastic products fully respect reuse, repair and recycling needs and more sustainable materials are developed and promoted. The hope is that this will deliver greater added value and prosperity in Europe and boost innovation as well as curb plastic pollution and its adverse impacts. The strategy presents key commitments for actions at EU level, which include that all plastics packaging placed on the EU market is either reusable or can be recycled in a cost-effective manner by 2030; that more than half of plastic waste generated in Europe is recycled by 2030; that sorting and recycling capacity has increased fourfold by 2030 compared to 2015; that demand for recycled plastics in Europe has increased four-fold and that substances that hamper recycling processes have been replaced or phased out [10].

The strategy cites a few scientific studies with regard to the potential annual energy savings that could be achieved from recycling all global plastic waste [68] and very large quantities of plastic waste being leaked into the environment from sources on land and at sea, generating significant economic and environmental damage [69]. Two reports from the EU Commission’s JRC are also cited. One for the cost of litter to EU fisheries, which was estimated to be about 1% of total revenues from catches by the EU fleet [70]. The other for single-use plastic items being among the items most commonly found on beaches, representing an estimated 50% of marine litter [71]. Finally, the Ellen MacArthur Foundation has published two reports on rethinking the future of plastics. Catalysing actions under the theme The New Plastics Economy was cited for information when it comes to e.g., value reduction of plastic packaging material [72]; collection and sorting systems to improve economics of plastic recycling; and design improvements to halve the cost of recycling plastic packaging waste [73].

The European Strategy for Plastics in a Circular Economy notes uncertainty about treatment of plastic waste abroad, market outlets and profitability for recycled plastics and lack of information on contamination by possible presence of chemicals of concern. The JRC report “Harm caused by Marine Litter” also points to some uncertainties. First of all, it states that the “General conclusions highlight that understanding the risks and uncertainties with regard to the harm caused by marine litter is closely associated with the precautionary principle. […] and to provide an evidence base for the various actions needed to be implemented by decision-makers”. It mentions that there is uncertainty present at each stage of an environmental risk assessment. Important uncertainties summarized in the report include poor understanding of how evidence of effects from localized studies could be extrapolated to larger spatial scales; that it is not possible to link evidence of the substantial numbers of individuals affected by marine plastics litter to negative effects on populations for most affected species; and relative importance of plastics as a vector for transport of chemicals from sea water to the food web.

The reports from the Ellen MacArthur Foundation [72, 73] do not mention uncertainty specifically besides noting that there are uncertainties about the potential consequences of long-term exposure to substances found in today’s plastics, about their combined effects and about the consequences of leakage into the biosphere as well as the uncertainties related to long-term risks of plastic packaging being recycled in multiple closed loops. With regard to the precautionary principle, Ellen MacArthur Foundation [73] states that regulators are often driven by the precautionary principle and potential cost to society in contrast to businesses that anticipate reputational risks and aim to capture potential economic value.

### Scientific foundation for the plastic waste amendments to the Basel Convention

In 2019, at the fourteenth meeting of the Conference of the Parties to the Basel Convention (COP-14), a series of plastic waste amendments were adopted. These became effective as of 1 January 2021. The Convention was revised with the objectives of enhancing the control of the transboundary movements of plastic waste and clarifying the scope of the Convention. Specifically, Annexes II, VIII and IX [11] were revised so that plastic waste falls into three categories under the Basel Convention, namely single polymer uncontaminated plastic waste, plastic waste requiring special consideration, and hazardous plastic waste.

In Annex VIII, plastic waste was inserted clarifying that it is presumed to be hazardous whereas Annex IX clarifies the types of plastic waste that are presumed not to be hazardous. The latter includes a group of cured resins, non-halogenated and fluorinated polymers, provided the waste is destined for recycling in an environmentally sound manner and almost free from contamination and other types of waste. It also includes mixtures of plastic waste consisting of polyethylene (PE), polypropylene (PP) or polyethylene terephthalate (PET). Annex VIII is subject to the Prior Informed Consent procedure (PIC) and Annex IX is not. Finally, a new entry Y48 was inserted in Annex II which covers plastic waste, including mixtures of such wastes unless these are hazardous or presumed not to be hazardous.

The amendments implemented to the Basel Convention were originally proposed by the Government of Norway (see [74–76]). The explanatory note by Norway on the proposed amendments does not contain any scientific argumentation, but notes that”…the discharge of non-hazardous solid plastic waste into the environment causes problems globally in the form of marine litter and microplastics and that a distinction needs to be made between hazardous plastic waste already covered by the PIC procedure, problematic streams of plastic waste that should be made subject to the prior informed procedure (Annex II-waste), and uncontaminated, pre-sorted plastic materials for recycling, prepared to a specification and suitable for immediate recycling. The latter group are less likely to pose environmental risks as a result of transboundary *movements…*” [75].

Norway had already suggested the removal of solid plastic waste from Annex IX to the Basel Convention under COP-11 [77]. In the application to amend Annex IX, Norway provides a summary of the reasons for the proposed changes. They highlight that 80–85% of all marine litter is plastic, which results in environmental and economic harm. They further address the extensive trade with plastic waste as an issue, since most of the marine plastic litter originates from poor waste handling.

Furthermore, it states that improving waste collection and management presents the most urgent solution to reduce plastic inputs, especially in developing economies. To support this, Norway cites the EC "A European Strategy for Plastics in a Circular Economy" for about half of the plastic waste collected is sent abroad where uncertainty remains over its treatment [10]. Finally, they argue that plastics are both persistent in the environment and have harmful impacts. The reasons are supported by the two reports conducted for UNEP. UNEP/EA.2/INF/23 [78] addressing marine plastic pollution and the link between research and policy, and UNEP/EA.3/INF/5 [79] focusing on combating marine plastic litter and microplastics.

The report termed UNEP/EA.3/INF/5 [79] provides scientific foundation for combating marine plastic litter and microplastics. The report states that much of the plastic waste that ends up in the oceans is a result of mismanagement. This is supported by the second UNEP report cited, which is termed UNEP/EA.3/INF/23 [78], which addresses the loss of solid plastic waste to the environment. The latter UNEP report, which was published in 2016, refers to Jambeck et al. [69] which again documented that between 4.7–12 million tons of plastic waste is lost to the marine environment each year from land-based sources. The Norwegian proposal furthermore draws upon a UNEP report made by the GESAMP Working Group 40 which consisted of some of the most recognized researchers working within marine plastic pollution [78]. The GESAMP Working Group 40 focused on assessing sources, fate and effects of plastics and micro-plastics in the marine environment, and concluded that ecological, societal, and economic impacts of macroplastics pollution have been well documented, and that precautionary measures are well justified since large uncertainties will remain for some time, which will prevent the generation of full risk assessments. The main uncertainties relate to exposure scenarios where sources are well documented, but the actual quantification of their importance remains uncertain. The UNEP report prepared by the GESAMP Working Group is further used as scientific foundation for stating that leakage of plastics into the ocean can occur at all stages of the production-use-disposal cycle, especially due to inadequate wastewater and solid waste collection and management.

The “Report on possible options available under the Basel Convention to further address marine plasti litter and microplastics” (UNEP/CHW/OEWG.11/INF/22) [80] mentioned by Norway was originally included in the work program of the Open-ended Working Group (OEWG) at COP-13. The report is a list of policy activities and does not in itself provide any scientific foundation for actions. The report again refers to work conducted by the GESAMP Working Group 40 as well as information from the UNEP’s Clean Seas Campaign. The report furthermore clarifies the scope of the Basel Convention and obligations, policies and guidelines related to and of relevance for plastic disposal and waste. Also, public awareness and education and sharing good practices and information are noted as being an important manner in which to address plastic pollution. Finally, the report lays out options under the Basel Convention to further address marine plastic litter and microplastics.

### Scientific foundation for the European single use plastics directive

In June, 2019, the EU Commission presented the “Directive (EU) 2019/904 of the European Parliament and of the Council of 5 June 2019 on the reduction of the impacts of certain plastic products on the environment” also known as the Single Use Plastics Directive (SUPD). The Directive originates from a broader approach in the EU to tackle plastic pollution in the environment combining the “European Strategy for Plastics in a Circular Economy” of 2018, the “Circular Economy Action Plan” of 2015 (since then the “New Circular Economy Action Plan” has been adopted in 2020) and the revised “Waste Framework Directive*”* of 2018. These three legislations incorporate plastic materials in terms of product design, recycling and targets for local waste management, while the SUPD aims to build upon these efforts and focus on prevention of marine plastic litter by market restrictions and having the producers pay for cleaning up [81]. Another highlighted motivational force for the Directive was the UN Sustainable Development Goal (SDG) no. 14 ‘Life below water’ which demands prevention and reduction of marine litter for future sustainable development and requires regulations in place to combat marine pollution [82]. Prior to the adopted legislation in 2018, an IA [81] was published by the EC addressing the urgency for EU action while considering the implications of proposed available solutions. The policy initiative target selected single use plastic (SUP) products and fishing gear based on monitoring data scrutinized in the IA.

The IA [81] consults the available data at EU level from experts and the consultancies Deloitte and Eunomia for assessment and modelling of policy options. The IA heavily relies on the work of the JRC Technical reports on marine litter occurrence and abundance in Europe. It states that “the best information comes from beach counts” and refers to the monitoring work by JRC Technical Group of Marine Litter Activities. Consultations with important stakeholders such as industry, members of the public, scientists, NGOs and government representatives from Member States and in the EU have fed into the overall development and direction for the IA on how to best and most effectively reduce plastic waste from SUP. When narrowing the number of plastic products down to 10 for the SUPD, the JRC Technical Report: Top Marine Beach Litter Items in Europe [71] appears as the underlying foundation. Based on the MSFD Master List Categories of Beach Litter Items the JRC report identifies 251 different types of litter. These were divided into plastic categories and finally listed as the top 10 items covering 86% of all SUP occurring on the European beaches and 77% of the plastic items found generally. In the JRC report, the top beach litter items of 2016 are derived from monitoring data produced by a broad collection of scientific projects, NGO reports and national authorities [71]. Use of different monitoring protocols across studies influence reporting methods and create obstacles for synthetizing data. However, it fortified the MSFD TG Marine Litter to issue a call in 2017 for available beach litter datasets across Europe in the identification of the top litter items using the ‘total abundance method’ which provides total numbers of each litter type for each survey at each beach for a certain time period [71]. The innate methodological variability and the use of beach monitoring as representative for the general state of plastic pollution at sea and on land are based on arguments of possible transportation pathways and accounted for in the IA. However, Syberg et al. [83], find that other nature compartments may be more relevant than beaches to include when monitoring plastic pollution in the environment. The top 10 list of SUP products are thus ranked by abundance on the European beaches and not by a risk-based approach accounting for environmental, economic, or societal harm as there is no existing risk-related litter ranking list available. Data quality variability in relation to monitoring data are inevitable as debris quantification is dependent on a series of site-specific conditions, methodical observation variance and lack of quality assurance [71].

Throughout the IA the arguments for the state of the environment and stress of plastic pollution are backed by peer-reviewed sources which represent the current research at the time [81]. However, highlighted studies in the section “Evidence used in the impact assessment” dates back approximately two decades, e.g., Jakus et al. [84] and Tiller et al. [85], both concerning household recycling, which shows a potential risk of excluding newer and more relevant inputs.

### Scientific foundation for the European ban on intentionally added microplastics

As part of the EU’s plastics strategy, aiming at changing how plastic products are designed, produced, used and recycled, the EC assessed the scientific evidence for taking regulatory measures to address releases of intentionally added microplastics for all types of consumer and professional use products in 2017 [86–88]. As a result, the European Chemicals Agency (ECHA) drafted a restriction proposal (restriction dossier) addressing these in early 2019. The restriction proposal was released for public consultation from March to September 2019 with a subsequent scrutiny and approval by the ECHA’s risk assessment committee (RAC) and committee for socio-economic analysis (SEAC). The combined final opinion of RAC and SEAC was published in February 2021. Currently, the EC is preparing a draft amendment to the Annex XVII (draft restriction), which will need to go through a discussion, including voting, with the member state competent authorities, and scrutiny by the European Council and the European Parliament before the restriction proposal can be adopted [86]. The restriction of intentionally added microplastics is expected to reduce the release of these by 70 to 95%, equalling approximately 400,000 to 500,000 tonnes of microplastics, over the span of 20 years [13, 87, 89]. Importantly, the restriction proposal does not seek to restrict the use of polymers in general but intends to restrict placing microplastics on the market on their own or in mixtures for uses that inevitably results in environmental releases; introduce labelling requirements for uses that do not result in inevitable releases; and to introduce reporting requirements to safeguard the quality of information available to assess potential future risks [13].

To establish a scientific knowledge-base, the EU funded a substantial amount of research projects related to microplastics in the environment years prior the restriction proposal. Of January 2016, as part of the Oceans Joint Programming Initiative, four research projects were launched with a total budget of €7.7 million [13]. Thus, the restriction proposal builds upon initiatives taken at least half a decade ago.

By observing the restriction proposal process, it is evident that the EC and the ECHA have taken several steps to account for the scientific state-of-the-art. As mentioned, the proposal was enabled after scrutiny of scientific evidence highlighting that microplastics constitute a potential risk to human health and the environment. The Annex XV restriction report prepared by the ECHA writes that it is “…setting out the main evidence justifying the proposed restriction…” clearly indicating that the restriction proposal builds upon “evidence” [13]. E.g., by April 2018, the EU Commission’s Group of Chief Scientific Advisors requested a review of evidence on microplastics in nature and society for scientific advice on microplastics pollution by the Science Advice for Policy by European Academies (SAPEA). Thus, the restriction proposal draws parallels to the data gathered in the report by the SAPEA, which was published simultaneously to the restriction proposal itself [13, 14]. The report by SAPEA stresses that microplastics are omnipresent and that there are several important scientific knowledge gaps that need to be addressed [14]. In relation to risks it is interesting to note that the SAPEA report does not to the same extent as ECHA, identify a risk of microplastics. The SAPEA report concludes that “The best available evidence suggests that microplastics and nanoplastics do not pose a widespread risk to humans or the environment, except in small pockets” [14]. Further, the ECHA based the restriction proposal directly on scientific evidence as they refer monitoring data and numerous scientific studies by independent research groups, member state competent authorities, the EC JRC and others such as the joint Group of Experts on the Scientific Aspects of Marine Environmental Protection (GESAMP) and use these for the basis of their arguments and claims. As an example, the ECHA refers to four studies for why they assume no (bio)degradation of microplastics during wastewater treatment, including three scientific papers and a report commissioned by the EC [13].

As part of the restriction proposal process, the proposal was subject to scrutiny by RAC and SEAC. Especially, the work by RAC is deeply rooted in science, as risk assessment builds upon scientific evidence for hazards and exposure. Due to the complexity of the hazards associated with microplastics, the risk assessment of RAC is based on three different approaches: the classical (eco)toxicological risk assessment establishing evidence for the predicted no-effect concentration (PNEC) and the predicted environmental concentration (PEC) with a subsequent evaluation of the risk quotient (PEC/PNEC); a non-threshold approach based on a persistent, bioaccumulative, toxic or very persistent, very bioaccumulative (PBT/vPvB) perspective. This implies that no ‘safe’ environmental concentrations can be established; and a ‘case-by-case’ assessment addressing the extreme persistence of microplastics in the environment [87]. The traditional risk assessment approaches were based on both scientific data and grey literature. It stresses that most data available is not specifically for intentionally added microplastic. Further, it critically analyses: “(i) key review papers on the topic (both from the peer reviewed and grey literature) and (ii) the most influential studies/articles published in the scientific literature…” and assesses their individual relevance and reliability [13]. Further, it recognises that many of the most influential studies are non-standardized. The non-threshold approach highlights the similarities between PBT/vPvB-substance properties and the properties of microplastics. Especially it is stressed that microplastics holds the potential “to accumulate within environmental compartments and biota, transfer between trophic levels, and the fact that they are practically impossible to remove from the environment” [13]. Importantly, in the restriction report, it is stressed that the PBT/vPvB approach conducted is not based on the currently available information as the traditional concepts of bioaccumulation and biomagnification might not be applicable for polymers on a molecular level [12]. In the ´case-by-case’ approach scientific data on microplastic degradation processes are scrutinised highlighting that degradation rates of different polymers vary from readily biodegradable to non-degradable. Also, it is stressed that there is a correlation between decreasing surface area and degradation rates and that degradation rates of larger plastic particles might represent a worst-case scenario.

The restriction proposal report refers to the Registration, Evaluation, Authorisation and Restriction of Chemicals (REACH) annex I which outlines how to conduct chemical risk assessments. The three approaches are also implemented in the annex XV restriction report and indicate that multiple scientific approaches have been applied [13]. According to RAC and SEAC, their work should be seen as complementary to the work by SAPEA and stresses that it was conducted independently of each other [89].

The ECHA considers the restriction of intentionally added microplastics to be both implementable and enforceable, even though harmonised analytical methods to detect microplastics in consumer products are not yet established. Also, methods for analysis of, and criteria for (bio)degradable plastics still needs further research. The argument behind, is that existing test methods are still applicable and can be readily applied to test for presence of microplastics in mixtures [13], which illustrate that ECHA have assessed the scientific, analytical and technical method available. Further, in the restriction report it is evident from the extensive use of references to the scientific literature that it is well-founded in science.

The work by SEAC entails an assessment of the socio-economic impacts of the proposal, including e.g., impacts on different sectors (agriculture, cosmetic, medical etc.). SEAC concluded that the restriction proposal is the most appropriate measure to handle the risks concluded by RAC, considering the socio-economic benefits and costs.

The restriction proposal report of intentionally added microplastics stresses that “risk assessment of microplastics is complicated by the current uncertainties apparent in relation to hazards, fate, exposure and risks” [13]. It addresses these uncertainties throughout the report but it also holds a separate section reflecting upon the uncertainties identified and the assumptions made. It justifies the use of the non-threshold based approach to risk management with the prevalent uncertainties mentioned. The IA assumes that biodegradable microplastics can substitute all non-degradable ones. The restriction report specifically stresses that a re-evaluation of the socioeconomic impacts of banning the non-degradable microplastics are required if the assumption is not met. Likewise, the implications of the uncertainty and assumptions made are addressed through sensitivity analysis taking ‘worst case’ values into consideration.

## Discussion

### Extent and topicality of the scientific evidence behind plastic policy initiatives

**Fig. 1:**
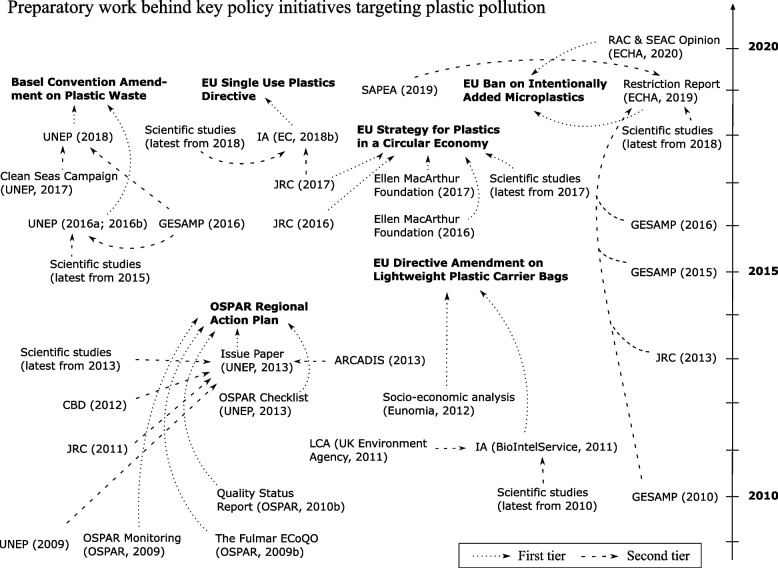
Historical view of the main pieces of the preparatory work that provides the knowledge foundation for six key policy initiatives targeting plastic pollution. ‘First tier’ documents constitute preparatory work for the initiatives and can be based on ‘second tier’ documents. Abbreviations: CBD: Convention on Biological Diversity; ECHA: European Chemicals Agency; EcoQO: Ecological Quality Objectives; EU: the European Union; GESAMP: Joint Group of Experts on the Scientific Aspects of Marine Environmental Protection; IA: Impact Assessment; JRC: Joint Research Centre; LCA: Life Cycle Assessment; SAPEA: Science Advice for Policy by European Academies; UNEP: United Nations Environment Programme

When diving into the preparatory work of the six policy initiatives targeting plastic pollution analyzed in this study, it appears that scientific evidence is generally present, and to a large extent for many initiatives. As it appears from Fig. 1, all the initiatives are supported by scientific articles and reports. Scientific articles were cited in the preparatory work and used to provide knowledge when it comes to e.g., plastics litter sources (OSPAR), energy savings (Plastic Strategy), and marine litter occurrence and abundance in Europe (SUPD). One article, Jambeck et al. [64], recurred and was cited in both the preparatory work of the Basel convention amendment, the European Strategy for Plastics in a Circular Economy and the Single-Use Plastics Directive where it provided evidence of leakage of plastics into the environment. Scientific reports prepared by JRC, UNEP and GESAMP were also found to be part of the knowledge foundation for most the policy initiatives, indicating a strong evidence base. For all the policy initiatives, except for the European Restriction Proposal on Intentionally Added Microplastics, reports by consultancies are also part of the science base underpinning the initiatives. Figure 1 shows that the scientific foundation for the policy initiatives seems to generally include the current knowledge available at the time of entry into force. All initiatives, except for the amendment of the EU Directive regarding lightweight plastic carrier bags, refer to knowledge that is published only a year prior to the initiation of the initiative. In the preparatory work behind the OSPAR Action Plan it is also claimed in a more general sense that “current scientific knowledge and monitoring results” are considered. Another initiative that clearly applies recent scientific knowledge is the EU restriction proposal on a ban on intentionally added microplastics. The restriction proposal refers to the SAPEA report [14] that was published right up to the release of the restriction proposal and includes a review of the evidence when it comes to microplastics in nature and society.

### Litter monitoring, ecosystem-based approach and risk assessment

Marine litter monitoring data was found to contribute to the evidence base for 4 out of the 6 policy initiatives. This includes among others the SUPD and the European Strategy for Plastics in a Circular Economy that both refer to the technical report by JRC on Top Marine Beach Litter Items [66]. The OSPAR Action Plan also heavily relies on monitoring data, including especially results from OSPAR Litter Monitoring [48] and the monitoring of marine litter in the stomachs of fulmars as part of the EcoQOs. This monitoring data and the quality report published in and around 2010 were also the precursor to the inclusion of descriptor 10 on marine litter in MSFD and the subsequent wave of policy actions tackling plastic pollution. The fact that these initiatives are underpinned by extensive marine litter monitoring highlights that monitoring appears to be one of the central scientific drivers behind the societal actions on plastic pollution.

In the 1990’s OSPAR adopts the so-called ecosystem-based approach as a way of protecting the marine environment through the integrated assessment and management of human activities. This approach is therefore also enshrined in the OSPAR Action Plan [43] and intends to “…identify and take action on influences which are critical to the health of the marine ecosystems, thereby achieving sustainable use of ecosystem goods and services and maintenance of ecosystems” [3]. The ecosystem-based approach has been supported by many scientists as an approach to management of the sea [90]. Back in 1999, Sherman & Duda [91] presented the ecosystem-based approach as a paradigm shift where assessment and management of coastal waters moved away from a short-term perspective to long-term management and spatially from smaller to larger scales, thereby considering ecosystems more broadly. The authors pointed to the approach as especially relevant when it comes to managing large marine ecosystems. The EU frameworks such as the Water Framework Directive from 2000 and the MSFD from 2008 also apply the ecosystem-based approach and thereby differ from the “end-of-pipe”/”single-issue” directives such as the EU directives on drinking water and birds and habitats.

### Uncertainty and precaution

**Fig. 2:**
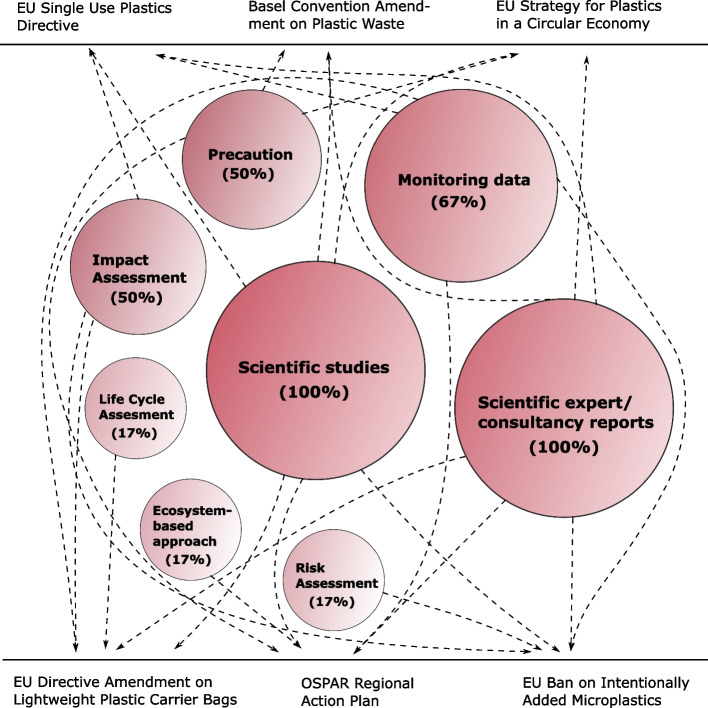
Type of scientific evidence, tools and principles in the preparatory work underpinning six key policy initiatives targeting plastic pollution. Arrows indicate which types have been applied in the preparatory work of each of the policy initiative(s). The size of the circles and percentages reflect the quantity of the investigated policy initiatives that apply each type of scientific evidence/tools/principles (bigger circle = greater application). The categories can overlap, e.g., many of the reports conducted by scientific experts or consultancy organisations are based on scientific studies and/or monitoring data. Abbreviations: EU: European Union

Elements of uncertainty exist within most of the scientific evidence that underpin the policy initiatives investigated in this study – which is not surprising, as there is always uncertainty in science. Going back to the earliest initiative analysed, the OSPAR Action Plan, the knowledge base includes a list of gaps when it comes to assessing ecological impacts and sources of marine litter. These knowledge gaps are outlined in one of the main documents underpinning the action plan, namely [43], which also includes a direct reference to the precautionary principle, thereby justifying actions despite of uncertainty. The precautionary principle is enshrined in the Treaty on the Functioning of the European Union [92] making it a fundamental aspect of European environmental regulation. In the report from JRC [70] supporting the European Plastics Strategy, it is stated that uncertainty is present at each stage of an environmental risk assessment, and it is recognized that the precautionary principle is closely linked to the understanding of risks and uncertainties when it comes to the harm caused by marine litter. The report further argues that when applying the precautionary principle in relation to marine litter, a threshold of harm or acceptability is essential. According to the report, this threshold of harm is very low when it comes to marine litter, because the Descriptor 10 of the MSFD is reached when “marine litter does not cause harm to the coastal and marine environment,” thereby activating the use of the precautionary actions [8, 70]. For three of the six policy initiatives analysed, precaution seem to be part of the foundation for taken action (Fig. 2). Precautionary measures are defended in the GESAMP report [78], supporting the Basel Convention amendment on plastic waste by its recognizing that microplastic pollution impacts are well documented and that great uncertainties are likely to continue to exist.

Since some of the policy initiatives analysed date years back, some of the knowledge gaps present at the time of implementation may have been fully or partly addressed since then due to ongoing research development. UNEP [78], one of the reports that constitutes the scientific foundation for the Basel Convention amendment on plastic waste, points to uncertainties when quantifying the relative importance of different plastic sources for determining exposure scenarios. Research within this field is still evolving, e.g., by Lebreton & Andrady [93] that studied projections of generation of global mismanaged plastic waste and its potential to reach the marine environment. Their estimates indicate that the load of mismanaged plastic waste will continue to be high in Africa and Asia in a business-as-usual scenario and that rivers are important pathways of plastic litter to the ocean.

As mentioned earlier, it is evident that monitoring data has played an important role as scientific foundation for initiatives targeting plastic pollution. Monitoring data constitutes the main basis for the selection of the SUP items included in the SUPD. This approach focuses on data for plastic item abundance and does not account for the environmental harm that the different items may cause. This is also highlighted in one of the documents [43] underpinning the OSPAR Action Plan, which notes that the plastic item’s impact is not accounted for, when considering the amounts of items. Uncertainties related to monitoring data are associated with the different reporting frequencies used as highlighted in the background documents to the OSPAR Action Plan [43] and the use of different monitoring protocols as highlighted in the background documents to the SUPD [71]. There may also be uncertainties when it comes to the representation of the environmental compartments in which monitoring is conducted. This is pointed out by Syberg et al. [83], arguing that beaches may not be the most contaminated ecosystem compartment, and thus not represent a worst-case scenario regarding concentrations of plastic pollution.

Some of the policy initiatives examined are based on estimations that are also associated with some uncertainties. One of these is the Directive amendment regarding lightweight plastic carrier bags, where the underpinning LCA helds uncertainties when it comes to e.g., consumer behavior, landfill conditions and method of waste combustion [62]. Similar uncertainties are identified in the restriction proposal report of intentionally added microplastics [13] that in general states that risk assessment of microplastics are challenging due to uncertainties when it comes to hazards, fate, exposure and risks. A list of assumptions is made, e.g., when it comes to the use of biodegradable microplastics as substitutions for non-degradable ones, and it is clearly stated that re-evaluation is required if the assumption does not hold.

### Revisiting the EC’s Communication for ‘Better Regulation’

Are plastic pollution policies evidence-driven? As mentioned in the introduction, the EC outlines in their Communication [29, 30] that one of the principles for ‘Better regulation’ is the use of scientific evidence to: describe the problem, understand causality and intervention logic and finally to assess impact. When it comes to the first pillar ‘description of the problem’ in the preparatory work of the plastic initiatives, it seems that the problem of plastic pollution is described by use of scientific evidence for all the initiatives (Table 1). The problem is described to the extent possible by reference to state-of-the-art knowledge on the area, and with the acknowledgement that knowledge gaps and uncertainties exist. For the two other pillars; ‘causality and intervention logic’ and ‘assessing impact’, there seem to be more variation (Table 1). Causality and intervention logic seem to be addressed for most of the policy initiatives, e.g., in the amendment to the BASEL convention and the Directive on light weight plastic carrier bags, where it is stated in the preparatory work that policy strategies are evaluated in relation to their effect. Furthermore, the OSPAR Action Plan applies the ecosystem-based approach which considers ecosystems dynamics in a holistic way. For initiatives that rely on litter monitoring there seem to be a link between the plastic products subject to the regulations and the dominant litter types and sources identified. As previously noted, it can be discussed how representative the counts are as well as whether litter abundance ranking of plastic types is the best foundation for regulatory prioritisation when these rankings are not risk-related. It is not fully clear whether all the policy initiatives analysed here are subject to assessment of the impact of the initiative, before and/or after its application. The directive amendment on light weight plastic carrier bags, the SUPD and ECHA’s restriction proposal on intentionally added microplastics were all subject to IAs before their application (Fig. 2). Certain types of post evaluation are also required. For the directive amendment on light weight plastic carrier bags it is required to assess the effectiveness of certain measures. Similarly, Article 15 in the SUPD requires that the EC carries out an evaluation of the directive (in 2027), e.g., when it comes to the need to review the list of SUP products and feasibility of binding collection rates. In ECHA’s restriction proposal it is also stated that re-evaluation of e.g., the socioeconomic impacts are required, if it turns out that the assumptions made in it are not met. The NEAES, that the OSPAR Action Plan builds upon, has recently be reviewed as part of the adoption of the OSPAR North-East Atlantic Environment Strategy 2030. In the strategy, reduction of marine litter is still a central element, aiming to e.g., “*Prevent inputs of and significantly reduce marine litter, including microplastics, in the marine environment…”* [94]. Our study also indicates that especially the two EU Directives included in the study score high when it comes to including scientific evidence in the preparatory work (Table 1).

**Table 1:** Use of scientific evidence in preparatory work of key policy initiatives targeting plastic pollution according to the principles of European Union’s Communication on ‘Better Regulation’ for evidence-informed policy. + : Preparatory work based on scientific evidence; -: Preparatory work not based on scientific evidence; ± : Preparatory work partly based on scientific evidence. Scientific evidence is defined according to the ‘Better Regulation’ toolbox [38]

Principles for evidence-informed policy^a^ (right) and policy initiatives (down)	Use of scientific evidence to describe problem	Use scientific evidence to understand causality and intervention logic	Use scientific evidence to assess impact
OSPAR & HELCOM Action Plans on Marine Litter	+	±	±
EU Directive Amendment on Lightweight Plastic Carrier Bags	+	+	+
EU Strategy for Plastics in a Circular Economy	+	-	-
Plastic Waste Amendments to the Basel Convention	+	+	-
EU Single Use Plastics Directive	+	±	+
The European Ban on Intentionally Added Microplastics	+	±	+

^a^According to the European Union’s Communication on ‘Better Regulation’ [29, 30] and ‘Better Regulation’ toolbox [38]

It should be noted that following the EU’s principles for ‘Better regulation’ when it comes to use of evidence, is not necessarily a proof that a policy initiative is more efficient or effective. Listorti et al. [95] investigated the debate on EU’s ‘Better Regulation’ Agenda, including the aspects of evidence-driven policy-making. The study highlights that to ensure effective regulation, methodological guidance is needed when it comes to especially selecting and presenting evidence, as well as guidance on how to implement policies. Furthermore, it has been argued that despite the EC’s dedication to evidence-based policy-making, political choices still need to be made and political logics, tools and procedures are still observed in the policy-making process [96]. This is also acknowledged by the EC, stating that evidence is only one factor influencing decision-making within the complex social and political processes [29].

## Conclusions

It is a declared ambition in the EU that policy development should be evidence-driven [29, 30, 38]. We explored to what extent scientific evidence has driven the development of six key policy initiatives targeting plastic pollution – an interesting question since numerous plastic policies have developed in the past decade despite the presence of uncertainties related to the impacts of plastic pollution. Our findings reveal that the preparatory work of the plastic initiatives overall seems to consist of – or refer to – scientific evidence in the form of especially scientific articles and reports prepared by experts or consultancy organisations. The scientific articles and reports provide knowledge about plastic sources, ecological impacts of plastics and production and consumption patterns. They include overview of current knowledge, i.e., knowledge published near the time of application of the initiative. More than half of the plastic policy initiatives examined in the present study refer to litter monitoring data, such as beach litter counts, as also constituting the scientific base. One of these is the SUPD which uses beach litter counts as base for the selection of SUP products in the directive, thereby emphasizing environmental abundance of different plastics types, rather than the potential environmental harm of these. In addition to scientific articles, scientific reports and monitoring data, a rather diverse group of different scientific tools seem to have been applied when shaping the plastic policy initiatives, including risk assessment, IA and life cycle assessment. Despite the prevalent consideration and application of scientific evidence, there appears to be a broad recognition in the preparatory work of the initiatives that there is still a lot of uncertainty related to determining the harm of plastic pollution. In these cases, taking precautionary actions seems to be justified, recalling that the precautionary approach is supposed to be a fundamental principle of European environmental regulation and that the principle is mentioned in several of the initiatives. Overall, scientific evidence is accounted for when shaping policy initiatives targeting plastic pollution. The issue of plastic pollution is complex and still related to several uncertainties, which implies that policy initiatives must allow for flexibility and on-going evaluations to adjust to the evolving knowledge generation. On the other hand, it is also important that the scientific community provides the needed research to continue the science informed policy development. A lot of focus in the scientific literature has been on handling plastics in the end-of-life phase [97], even though it is commonly agreed that a research into all phases in the value chain is needed to provide the best scientific foundation for policy making in the future.

Finally, it is important to recognize that the many different types of evidence used to support policy initiatives also provide room for scientific support for policy initiatives that might be conflicting. This can have consequences for the negotiations of the forthcoming United Nations plastic treaty, and even prevent a successful process. So, in order to ensure the best scientific evidence for these negotiations it is thus important that future studies aim at generating transparency about scientific foundations for ongoing and future policy initiatives around the globe, thereby providing the best scientific foundations for these important policy decisions.

## Data Availability

Not applicable.

## Abbreviations

BIM: Bord Iascaigh Mhara
CoG: OSPAR Coordination Group
EC: European Commission
ECHA: European Chemicals Agency
EcoQO: Ecological Quality Objectives
EU: European Union
HDPE: High Density Poly Ethylene
IA: Impact Assessment
ICES: International Council for the Exploration of the Sea
ICG-ML: Inter-sessional Correspondence Group on Marine Litter
JRC: Joint Research Centre
LCA: Life-cycle Assessment
LDPE: Low density polyethylene
LLDPE: Linear low density polyethylene
MSFD: European Marine Strategy Framework Directive
NRC: National Research Council
OEWG: Open-ended Working Group
PBT: Persistent, Bioaccumulative, Toxic
PE: Polyethylene
PEC: Predicted Environmental Concentration
PET: Polyethylene terephthalate
PIC: Prior Informed Consent procedure
PNEC: Predicted No-effect Concentration
PP: Polypropylene
RAC: Committee for Risk Assessment
REACH: Registration, Evaluation, Authorisation and Restriction of Chemicals
SAPEA: Science Advice for Policy by European Academies
SDG: Sustainable Development Goal
SEAC: Committee for Socio-economic Analysis
SUP: Single use plastic
SUPD: Single Use Plastics Directive
TFEU: Treaty on the Functioning of the European Union
TG-ML: Technical Group on Marine Litter
UNEA: United National Environmental Assembly
UNEP: United Nations Environment Programme
vPvB: Very Persistent, Very Bioaccumulative
